# Psychological disturbances encountered by selected undergraduates studying at the University of Ruhuna, Sri Lanka during the Covid-19 pandemic: a cross-sectional study

**DOI:** 10.1038/s41598-023-47950-8

**Published:** 2023-11-23

**Authors:** T. H. M. Kaushani, E. B. Weeratunga

**Affiliations:** Department of Nursing, Faculty of Allied Health Sciences, University of Ruhuna, Galle, Sri Lanka

**Keywords:** Psychology, Health care, Health occupations, Medical research, Risk factors, Signs and symptoms

## Abstract

University undergraduates are increasingly recognized as a vulnerable population with a higher level of psychological disturbances. During the Covid-19 pandemic, universities closed all over the world, resulting in the psychological well-being of this population being severely affected across the globe. This study examined the prevalence of stress, anxiety, and depressive symptoms encountered by undergraduates of the selected five faculties at the University of Ruhuna in Southern Sri Lanka due to the Covid-19 pandemic, its associated factors, and the correlations between stress, anxiety, and depressive symptoms. An online, cross-sectional, descriptive study was conducted among undergraduates using pre-tested, self-administered questionnaires from the randomly selected five faculties: Allied Health Sciences, Engineering, Humanities and Social Sciences, Management and Finance, and Medicine. Undergraduates were invited to complete the Google Form. Socio-demographic details and a 21-item Depression, Anxiety, and Stress Scale (21-DASS) were used for data collection. Stress, anxiety, and depressive symptoms were evaluated using a Sinhala version of the 21-DASS scale. Ethical permission was granted by the Ethics Review Committee of the Faculty of Allied Health Sciences, University of Ruhuna. Among the 359 undergraduates, the majority were represented by females (62.1%). The mean age of the sample was 23.67 years (SD ± 1.6). Stress, anxiety, and depressive symptoms were found to be prevalent in 53.7%, 41.8%, and 63.8% of undergraduates respectively. Younger and female undergraduates had more impact on psychological issues than their counterparts. It revealed a significant positive correlation between anxiety and depressive symptoms (r = 0.646, *p* < .001), anxiety and stress (r = 0.868, *p* < .001), and stress and anxiety (r = 0.786, *p* < .001). Most undergraduates experienced considerable levels of stress, anxiety, and depressive symptoms during the Covid-19 as increasing stress, anxiety, or depressive symptoms, tend to increase all types of psychological disturbances. The development of mental health among undergraduates is essential and needs innovative strategies to improve the psychological well-being of undergraduates. The initiation of a stress management programme and expanded available counseling services are also important. Further studies are needed to be conducted on the extended topic of how the Covid-19 era is affecting the psychological well-being of undergraduates from different universities (state/non-state), locations, and different study departments.

## Introduction

Severe acute respiratory syndrome coronavirus 2 (SARS CoV-2) is the cause of Covid-19 which was an extremely contagious airborne disease from 2019 onwards^1,2^. World Health Organization (WHO) initially declared that this is a ‘Global Pandemic’ on the 30th of January 2020^3^, and an increase in confirmed cases and deaths was reported in Sri Lanka^4^. Due to Covid-19, all people had to practice lifestyle modifications such as wearing a facemask, washing hands continuously, using sanitizers, maintaining distance, etc.^5^ At the community level, mitigation efforts include banning travel, lock-down cities/countries, and travel restrictions to reduce the number of cases by reducing large gatherings^6^. Many countries employed a range of control measures like restricting daily activities, community screening, and promotion of safety measures to control the transmission of Covid-19 among humans^4,7–10^.

The closure of universities and education organizations directly affects the education of the student/undergraduate population in the world^11–13^ such as either postponing or canceling all events, workshops, conferences, and sports and shifting rapidly to the transition of various courses and programs from face-to-face to online mode while being confined to hostels or any kind of living place^13^ facing significant challenges such as disruptions of teaching and learning activities were influenced by the school and university sectors^14^. According to the United Nations Educational Scientific and Cultural Organization (UNESCO), the education of more than one billion students in 129 countries in the world has been affected due to Covid-19^15^.

University undergraduates are identified as a population with weak mental health and reported as a “very high-risk population” with a higher prevalence of mental illnesses such as depressive symptoms, anxiety, stress, etc., than the adult population; the psychological health of university undergraduates is considered internationally as an essential public health issue^2,14,16–20^. Stress is defined as “a particular relationship between the person and the environment that the person considers being taxing or exceeding his or her resources and putting his or her well-being at risk”^21^. Stress is not a novel problem for university students; they are a special group of people who are going through a critical transition period in which they are going from adolescence to adulthood, and this can be the most stressful time in one's life. Depression is defined as “a common mental disorder that presents with depressed mood, loss of interest or pleasure, decreased energy, feelings of guilt or low self-worth, disturbed sleep or appetite, and poor concentration”^22^. The past ten years have seen a rise in interest in researching university undergraduates' mental health, with university undergraduates showing a 24–34% incidence of depressive symptoms^23^. Anxiety is a behavioral, physiological, and psychological reaction^24^.

There were ample research findings that Covid-19 has impacted peoples’ emotions such as healthcare professionals, the general public, and patients, etc.^25–27^. Numerous research has confirmed that the Covid-19 crisis caused a high prevalence of stress and a moderate prevalence of anxiety and depression^28–32^. One study in China found that 53.8% of respondents had severe to moderate psychological disturbances in female undergraduates^15^. The private and academic/university lives among undergraduates in Saudi Arabia were affected severely by Covid-19 and reported the profound psychological impact on university undergraduates^33,34^.

As reported in some studies, human mental health and well-being are influenced by changing environmental factors such as Covid-19^2,14^. Almost all people must deal with the new normalcy of not only the disease itself but also the after-effects of the Covid-19 pandemic. Most university undergraduates have faced many challenges because of numerous unanticipated changes and a new normal, which has increased several psychological disturbances, particularly depression symptoms, anxiety, and stress during the Covid-19 pandemic^35^. Therefore, undergraduates must cope with their new lifestyle although they are at home and need to continue their ongoing education. Due to all circumstances, many undergraduates have reported that they have poor mental health^36^. Further, variations in daily routine such as lack of outdoor activity, interrupted sleeping patterns and social distancing have impacted the mental well-being of many undergraduates^16^. In this vulnerable population, mental health needs to be closely monitored during lockdown periods^16^. Due to such changes, undergraduates had to face many issues related to technology (e.g., connection and internet problems), new devices (smartphones/laptops, tab, etc.), and economic hardships^36^. Most educational institutes have been changed to remote/distance learning and online learning; due to such experiences, extra burdens such as low internet quality, no proper access to new technology, few resources at home, the trouble of managing available technical devices and learning aids when continuing their studies had impacted on their education^37^.

When compared to the global impact of Covid-19, Sri Lanka started the battle during the first few months of 2020. According to the emerging situation, the Government of Sri Lanka endorsed lockdown and travel restrictions as Western countries. In addition, Sri Lanka as a developing country is going through the most terrible economic crisis^38^ which influenced every aspect of the life of people. Therefore, most undergraduates in government universities live under many socio-economic constraints/hardships usually, and face difficulties in buying smartphones/devices, and internet facilities to continue their online teaching–learning activities which was a drastic change in the Sri Lankan education system not perceived before^18^. Sri Lankan state universities are located across the country and have their university sub-culture on factors such as history, geographical location, regionalism, university size, residential pattern, and political ideology^18^. Unlike the Western literature, a few researchers in Sri Lanka have been studying psychological problems and coping strategies encountered by undergraduates studying in some state and non-state universities. The distance-learning undergraduates at the Open University of Sri Lanka experienced nearly 51% psychological distress due to the distance mode of education while having low levels of anxiety and depression^39^. Some studies have reported psychological ill-being among undergraduates due to the combat of Covid-19^40^. However, there have been no published studies on psychological disturbances caused by the Covid-19 epidemic in undergraduates studying in the Southern province of Sri Lanka when compared with the Western province of Sri Lanka. The virulence of the disease was slightly lower in the Southern province to a certain extent than in the Western province comparatively and university academics in the Southern province were able to initiate some academic activities (e.g., online/virtual or physical) before the other universities in Sri Lanka.

The University of Ruhuna (UoR) is situated in the Matara district, Southern province of Sri Lanka. Therefore, this study aimed to investigate the psychological disturbances encountered by undergraduates of the selected five faculties at the UoR, Southern Sri Lanka due to the Covid-19 pandemic. This study aimed to examine the prevalence of anxiety, stress, and depressive symptoms among undergraduates, its associated factors, and correlations of anxiety, stress, and depressive symptoms. The study findings were an overview of undergraduates’ mental health of the UoR which had not been encountered earlier due to the pandemic situations and new normalcy. Further, this study would assist in identifying remedial action to reduce or prevent such psychological imbalances and would help to increase the academic performances and student support services of undergraduates when there are occurrences of future pandemics. The research will be useful for future policymakers to establish welfare events and psychological support services such as counseling for undergraduates not to suffer from psychological ill-being.

## Material and methods

### Study designs, setting, and participants

A descriptive, cross-sectional study design was used to investigate the psychological disturbances (e.g., distress, anxiety, and depressive symptoms) encountered by undergraduates of the selected five faculties, at the UoR, Matara, Southern Province in Sri Lanka due to the Covid-19 pandemic. The UoR is the only state university in the Southern Province and it consists of ten faculties, some of them are located away from the main premises and are in different study settings like Galle [e.g., Faculty of Allied Health Sciences (FoAHS), Faculty of Engineering (FoE), and Faculty of Medicine (FoM)] and Matara [e.g., Faculty of Humanities and Social Sciences (FoHSS) and Faculty of Management and Finance (FoMF)]. The undergraduate population of the university is more than 10,000. This study was conducted from mid-June to August 2022. The study participants were undergraduates who were in 1st year to 4th year and studied in the -above-mentioned faculties of the UoR.

### Sample size and sampling procedure

The sample was calculated by using the following formula^41^ (n = Z^2^ P (1 − P)/d^2^ [n = size of the sample, Z = 1.96, P = anticipated population proportion, d = absolute precision required for the estimate to fall within given, percentage point of the proportion = 5%]. The sample size was calculated using the prevalence of the previous study (n = 383.62)^42^. After adding a 10% non-responsive rate, the final sample size was taken as 422; a stratified sampling procedure was applied (422/5 = 84) and 84 students were randomly selected from each faculty using a random table and incorporated the name register of the undergraduates. Further, among some students who studied in the 1st year to 4th year; nearly 21 undergraduates were selected each year to represent whole undergraduates in the faculty and taken as strata. Undergraduates received an electronic questionnaire via batch representatives and professional networks. An equal number of students from each faculty were sought for the survey.

Undergraduates who studied in the FoAHS, FoM, FoE, FoHSS, and FoMF, at the University of Ruhuna, could read and/or understand English or Sinhala, and were in good psychological well-being (i.e., who do not have psychological disorders or were taking treatments for psychological disorders) were considered as inclusion criteria and undergraduates who have studied in the other faculties in addition to the above-mentioned faculties and were unable to read and/or understand Sinhala or English were not recruited. Undergraduates who fulfilled the above criteria were selected using the above sampling procedure and invited to participate in this survey.

### Data collection tool

A self-administered questionnaire was prepared by authors, pre-tested using another ten undergraduates, and administered as an online survey due to the pandemic situation. This Google Form consisted of two parts; socio-demographic details (e.g., age, gender, faculty, academic year, and monthly parents’ income) and a 21-item DASS scale^43^. The internal consistencies for each scale of the original DASS scale were: depression 0.91; anxiety 0.84; stress 0.90; also, supported for convergent and discriminant validity^43^. In 2008, good-to-excellent internal consistency was obtained for three sub-scales (depression-0.96, anxiety-0.89, stress-0.93), and satisfactory criterion validity (r = 0.65)^44^. Also, in 2012, excellent test–retest reliability was achieved for 21-DASS (r = 0.71–8.81)^45^.

Sinhalese version of 21-DASS^46^ had reported satisfactory internal consistencies for depression- 0.83, anxiety-0.76, and stress-0.80. The validity was checked using a Patient Health Questionnaire (PHQ) and DASS anxiety, and obtained a positive correlation for three sub-scales (0.544, 0.473, and 0.333); the DASS stress scale had a positive correlation with PHQ (0.558). The factor structure of the 21-DASS was similar to the original scale and proved the confirmatory factor structure too. Every subscale consists of seven items and was used to calculate levels of depression (items 3, 5, 10, 13, 16, 17 and 21), anxiety (items 2, 4, 7, 9, 15, 19 and 20), and stress (items 1, 6, 8, 11, 12, 14 and 18). Every question of DASS-21 has four responses 0 to 3. The responses were; 0—didn’t apply to me at all, 1—applied to me to some degree or occasionally, 2—applied to me at a considerable level or frequently, and 3—applied to me a lot or frequently. The validated DASS-21 was used to examine depressive symptoms, anxiety, and stress., and the scoring method is presented in Table 1.

**Table 1:** Scoring method of stress, anxiety, and depression levels.

Severity levels	Stress	Anxiety	Depression
Normal	0–10	0–6	0–9
Mild	11–18	7–9	10–12
Moderate	19–26	10–14	13–20
Severe	27–34	15–19	21–27
Extremely severe	35–42	20–42	28–42

### Data collection procedure

Prior permission was obtained from the Vice Chancellor of the University of Ruhuna and the Deans of all faculties. Further, the respective e-mail addresses of undergraduates were received from assistant registrars of all faculties and invited to the selected undergraduates via an e-link. Socio-demographic details and answers of 21-DASS were collected using a web-based, self-administered questionnaire (Google Form); their voluntary consent was obtained after providing adequate facts via an information sheet (e.g., purpose, responsibilities of participants, potential benefits and risks to the participant, confidentiality and anonymity of data collection, and procedure and period of data storage). After reading the information sheet, a voluntary informed consent form appeared; once given the consent using an electronic format, undergraduates were able to answer e-questionnaires. The medium of the questionnaire was English or Sinhala and they had an opportunity to select the best choice of language. After filling in all questions, they had to submit a Google Form to the given e-mail of the first author. To ensure the privacy and accuracy of the data, an anonymous questionnaire was used for the survey, and data accessibility was in the custody of the authors.

### Data quality assurance

All probable interventions were taken to increase the quality of data during the whole process. The questionnaire was pre-tested as mentioned earlier before starting the data collection and then any necessary improvements were made. The purpose of the study and the nature of the study were informed to all participants via an e-information sheet. Participants had the right not to respond to the questionnaire and leave at any time without providing any clarifications. As only the principal investigator had the authority about data and data collection was only done by the principal investigator, confidentiality was ensured. Every undergraduate was identified using a code/serial number to protect their privacy and anonymity, their rights were respected, and confidentiality of information was strictly maintained. Before analysis, data were checked for consistency. Answers from the participants were accessible only to investigators, and confidentiality was protected using passwords employed to control the quality of data.

### Data analysis

Statistical Package for Social Science (SPSS) software was used to analyze data. Socio-demographic data were examined using descriptive analysis such as frequency and percentage, which can be summarized by using a graph/chart. According to the recommended cut-off values, the depressive symptoms, anxiety, and stress were divided into five categories: normal, mild, moderate, severe, and extremely severe^43^. Categorical variables were tabulated using the Chi-square test and association was checked using the Pearson correlation coefficient. All results were regarded as statically significant at *p* < 0.05.

### Ethical clearance

Ethical approval was obtained from the Ethics Review Committee of the Faculty of Allied Health Science, University of Ruhuna, Sri Lanka (Ref, no: 61.11.2021). All research procedures were carried out corresponding to the relevant guidelines and regulations by the Declaration of Helsinki. Necessary approvals were obtained from the authorities of the selected five faculties and the University of Ruhuna. Participants were invited using the Google Form and by an e-link due to the status of the country according to the health guidelines. Identification/serial numbers were given to maintain the anonymity and confidentiality of each undergraduate and therefore all responses were anonymous. Only the investigator had accessed the participant’s information. Voluntary participation was highly expected and written informed consent was undertaken from the study participants before the data collection. The rights of participants were respected, and the privacy, and confidentiality of information were strictly maintained while not taking identifying information.

## Results

### Socio-demographic details of the participants

Although 389 students agreed to participate, 359 students were included in the final analyses. Table 2 demonstrates the socio-demographic characteristics of the undergraduates. The mean (SD) age was 23.67 years (standard deviation (SD = 1.619)), and the majority were female undergraduates (62.1%).

**Table 2:** Demographic characteristics of the undergraduates (N = 359).

Demographic variables	n	%
Gender		
**Female**	**223**	**62.1**
Male	136	37.9
Name of the Faculty		
**FoAHS**	**102**	**28.4**
FoE	76	21.2
FoHSS	62	17.3
FoMF	61	17
FoM	58	16.2
Academic year		
**1st year**	**96**	**26.7**
2nd year	88	24.5
3rd year	84	23.4
4th year	91	25.4
Monthly income of parents (LKR)		
Below 20,000/=	67	18.7
Between 20,000/= − 39.999/=	69	19.2
**Between 40,000/= − 59,999/=**	**90**	**25.1**
Between 60,000/= − 79,999/=	60	16.7
Between 80,000/= − 99,999/=	27	7.6
Above 100,000/=	46	12.8

Frequency (n); percentage (%); LKR—Sri Lankan Rupees.

Higher values are in [bold].

The major ethnic and religious group was Sinhalese-Buddhist (data not shown). From the sample, an approximately equal proportion of undergraduates (1st–4th year) was represented. The majority of participants (income of parents of undergraduates) (n = 90; 25%) earned between LKR. 40,000 and LKR. 59,999 per month and 18.7% of participants had a lower income than LKR. 20,000.00.

### Prevalence of stress, anxiety, and depressive symptoms

#### Stress among undergraduates

**Figure 1:**
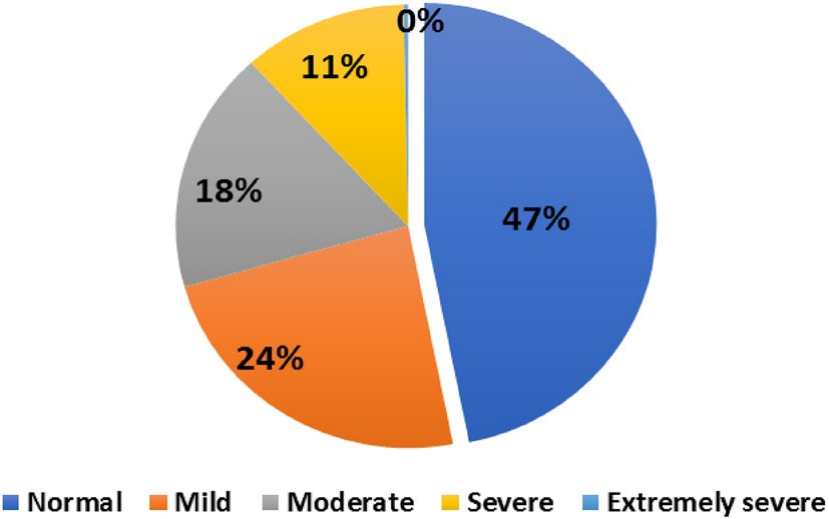
Stress distribution among undergraduates (n = 358).

Different levels of stress are exhibited in Fig. 1. Of the sample, many respondents (n = 168; 47%) showed a normal level of stress, followed by mild stress (n = 85; 24%), moderate stress (n = 63; 18%), severe stress (n = 41; 11%), and extremely severe stress (n = 1; 0%).

#### Anxiety among undergraduates

**Figure 2:**
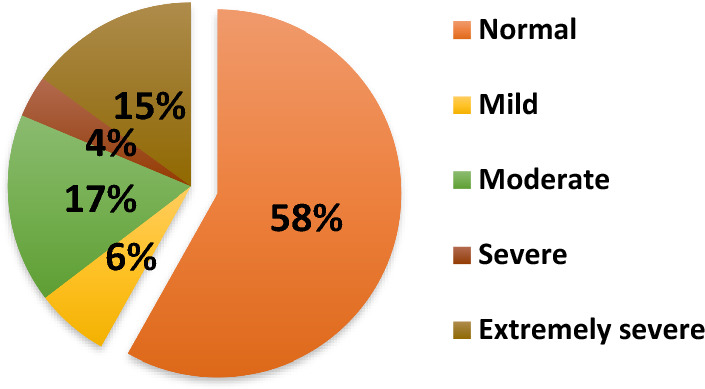
Anxiety distribution among undergraduates (n = 354).

As shown in Fig. 2, most undergraduates had a normal level of anxiety (n = 206; 58%) while only 15% of respondents (n = 53) reported extremely severe symptoms and 17% of undergraduates (n = 59) had a moderate level of anxiety.

#### Depressive symptoms among undergraduates

**Figure 3:**
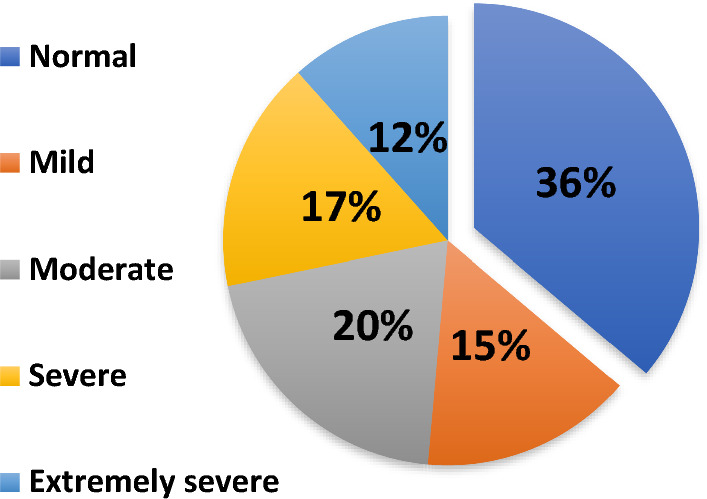
Depressive symptoms distribution among undergraduates (n = 354).

Overall, it was discovered that 37% (n = 131) of the subjects had increased (moderate to severe) depressive symptoms as presented in Fig. 3. From the sample, 12% of undergraduates (n = 41) had extremely severe levels of depressive symptoms while 36% (n = 128) were at a normal level.

### Factors associated with stress, anxiety, and depressive symptoms

In Table 3, associations between socio-demographic variables and different levels of psychological disturbances are exhibited. Undergraduates who were between the ages of 23 and 24 had significantly severe stress (*p* < 0.001). Considering the stress levels and faculties, FoHSS had a significantly higher number of participants with severe stress compared to other faculties (*p* < 0.001). However, gender (*p* = 0.06) and academic year (*p* = 0.233) did not show any significant association with stress levels.

**Table 3:** Correlations between socio-demographic variables and levels of stress, anxiety, and depressive symptoms.

	Categories/levels															
	Stress					Anxiety						Depressive symptoms				
Mean (SD)	13.05 (9.68)					8.15 (8.74)						14.38 (9.63)				
Variables	Stress (n = 358)					Anxiety (n = 354)						Depressive symptoms (n = 354)				
	N	M	Mo	S	ES	N	M	Mo	S		ES	N	M	Mo	S	ES
Age (y)																
≤ 22	49	24	12	5	0	60	6	19	0		5	43	11	15	15	5
23	35	33	14	**19**	0	50	10	13	1		**22**	25	14	21	**25**	**21**
24	40	9	5	**14**	0	38	3	6	**12**		9	27	12	16	4	9
≥ 25	54	19	32	3	1	58	4	21	**12**		**17**	32	17	20	**25**	6
*p value*	0.000**					0.000**						0.000**				
Gender																
** Female**	92	58	46	**26**	1	127	20	25	**9**	**37**		68	33	41	**43**	**33**
Male	76	27	17	15	0	79	3	34	4	16		60	21	31	16	8
*p value*	*0.06*					0.002**						0.008**				
Faculty																
FoAHS	57	16	23	4	1	57	16	23	4	1		41	21	17	**17**	6
FoE	40	16	8	12	0	40	16	8	12	0		32	10	24	1	9
FoHSS	18	19	9	**16**	0	18	19	9	**16**	0		18	5	9	8	**17**
FoMF	24	21	13	3	0	24	21	13	3	0		14	11	16	**17**	3
FoM	29	13	10	6	0	29	13	10	6	0		23	7	6	16	6
*p value*	0.000**					0.036*						0.000**				
Academic Year																
1st year	56	16	11	**13**	0	56	16	11	**13**	0		45	17	16	5	11
2nd year	44	22	13	9	0	44	22	13	9	0		34	11	18	19	6
3rd year	33	19	20	10	1	33	19	20	10	1		21	13	16	**21**	10
4th year	35	28	19	9	0	35	28	19	9	0		28	13	22	14	**14**
*p value*	*0.233*					*0.331*						0.044*				

Categories; N-Normal; M-Mild; Mo-Moderate; S-Severe; ES- Extremely severe. FoAHS-Faculty of Allied Health Sciences, FoE-Faculty of Education, FoHSS-Faculty of Humanities and Social Sciences, FoMF-Faculty of Management and Finance, FoM-Faculty of Medicine. **The 0.01 level of significance for correlation (2-tailed) and *0.05 level of significance for correlation (1-tailed).

Higher values in S and ES categories are in [bold].

Like stress levels, undergraduates who were aged 23, 24, and ≥ 25 years had a significant level of severe and extremely severe anxiety during the Covid-19 period (*p* < 0.001) (Table 3). Female undergraduates had a significantly severe and extremely severe level of anxiety than male undergraduates (*p* < 0.001). As for stress level, many undergraduates of FoHSS showed a significantly more severe anxiety level (*p* < 0.05) than other faculty undergraduates. Many undergraduates studied in the first year reported more severe anxiety than other academic years. Additionally, it was shown that 23 and ≥ 25 years of age undergraduates have significantly reported more severe and extremely severe depressive symptoms (*p* < 0.001) than other age groups (Table 3). More female undergraduates showed significantly severe and extremely severe levels of depressive symptoms than male undergraduates (*p* < 0.001). Also, undergraduates in FoAHS, FoHSS, and FoMF have reported significant severe and extremely severe levels of depressive symptoms (*p* < 0.001). Participants who were in the 3rd and 4th academic year showed a significant level of depressive symptoms (severe and extremely severe) than their counterparts (*p* < 0.05).

As reported, positive and significant correlations were found among each other psychological disturbances; stress and anxiety (r = 0.786; *p* < 0.001), stress and depression (r = 0.868; *p* < 0.001), and anxiety and depression (r = 0.646; *p* < 0.001); increasing stress, anxiety, and depression caused to increase in all reported psychological illnesses again.

## Discussion

### Prevalence of stress, anxiety, and depressive symptoms

This research examined the prevalence of stress, anxiety, and depressive symptoms experienced among university undergraduates at the selected faculties of the UoR, Sri Lanka, during the Covid-19 pandemic, its associated factors, and the correlations of those psychological disturbances. The prevalence of stress, anxiety, and depressive symptoms was 53%, 42%, and 64%, respectively in this study. Therefore, a higher percentage of undergraduates encountered stress, anxiety, and depressive symptoms in the current study.

The Covid-19 pandemic has significantly affected different types of university undergraduates worldwide, making them more susceptible to the emergence of several mental diseases like stress, anxiety, depression, etc., which are in line with some studies that reported higher prevalence^33,34,47–50^ and slightly lower prevalence^1,2,29,51–53^. Covid-19 and the lock-down caused different types of psychological problems among the Chinese population^10^; Arabian, Chinese, and Polish researchers found that undergraduates displayed mild to moderate anxiety symptoms (1–22%) but very low levels of severe anxiety symptoms (0–1%)^29,31,51,52,54^ compared to the current findings. According to another study from Malaysia, respondents suffered from mild to moderate anxiety levels (6%-20%) and 2.8% had severe anxiety levels^15^ but lower than the anxiety levels in the current study. However, some undergraduates had higher levels of anxiety (50–60%)^48,50^, and 18.1% of severe levels of anxiety in line with the current findings^50^.

In India, levels of anxiety and stress were higher during the Covid-19 pandemic than before^55^; 269 Indian medical undergraduates were evaluated for levels of anxiety and stress and reported 21.2% anxiety and 20.7% stress which was lower than this study findings^55^. A higher level of stress was found among Jordan undergraduates which was around 60% during the home-quarantine period and reported mild (15.5%), moderate (19.6%), severe (13.6%), extremely severe (10.0%) levels of stress^49^ and around 56% of stress was reported among Polish undergraduates^54^. Some undergraduates in the United States of America perceived an increased level of study‐related stress (e.g., due to related campus closure and shift to virtual classes) after the outbreak of the Covid‐19 pandemic^35^. A lower level of stress was found among undergraduates in France than among non-students who scored 33.1%^16^.

Depressive symptoms are a very common and widespread problem among undergraduates. In response to this stress, some undergraduates become depressed^19^. The past ten years have seen a rise in interest in researching undergraduates' mental health; a 24–34% incidence of depressive symptoms was reported in the very early period^23^. Another study conducted in one of the large universities in Saudi Arabia during 2012–2013, reported prevalence of depressive symptoms was 46% before the pandemic^56^ and was not much different from the current depression levels although studies were done with or without Covid-19. However, above 80% of depressive symptoms were evidenced in the Bangladesh undergraduates^50^ than the current findings and 78.7% had depressive symptoms in the Jordan study^49^. Severe levels of depression symptoms were in the range of 10–12%^50^. Not only the higher values, a considerable level of depressive symptoms was found among France and Arabic undergraduates (32% and 49%) due to Covid-19^16,33^ and lower depressive symptoms (10–20%)^31,52^. In 2020, 61.4% of undergraduates in Jordan (N = 1165) were diagnosed with moderate-to-severe depression^57^ and this was consistent with the current findings. As mentioned by Saraswathi et al.^55^ depressive symptoms had not changed during the pandemic whereas anxiety and stress increased due to the pandemic. Wang et al.^52^ also reported 12.2% of depressive symptoms which was very lower than the current depressive symptom levels. Among studied 3044 undergraduates in Saudi Arabia, 47.7% of individuals reported having severe depressive symptoms^34^.

Similar impacts were stated in Sri Lanka as well. Therefore, it is necessary to effectively report the psychological disturbances of Sri Lankan undergraduates due to Covid-19—how it could affect their academic performances; how this type of unexpected circumstances affect the very gentle personalities the young population have; and how their normal education which is not normal to them as previous is impacted. However, studies related to other undergraduates are scarce^40,58–60^ compared to the studies done among medical undergraduates in Sri Lanka^12,61–64^. Several research findings about the prevalence of psychological issues and factors associated with the Covid-19 disease among undergraduates in Sri Lanka were reported^7,40,58,59,62,63^. It was evidenced by a study conducted among children, adolescents, and youth in Sri Lanka^12^, that there were significant psychosocial stressors, and it may tend to develop more psychological morbidity among that population.

In some literature, lower levels of stress and anxiety were found. Covid-19 epidemic had a significant impact on medical students' psychological health (25.1%) and (1.6%) of students reported having anxiety and stress levels that were over the recommended levels^61^. One study done in Sri Lanka in 2020, including Indian and Russian undergraduates, reported that 3.16% of undergraduates experienced stress and 11.58% of undergraduates had anxiety due to Covid-19 which was remarkably lower than the current study^7^. Another finding of nursing undergraduates of KAATSU International University (KIU) reported the prevalence of anxiety and stress as 13% and 3%^65^ which were also much lower. In the first phase of the Covid-19 epidemic, 22% of students at the Sri Lanka Institute of Advanced Technological Education (SLIATE) showed mild anxiety, 7% showed significant anxiety, and just 2% showed severe anxiety. Thirty undergraduates were used in the study by Athukorala and Joniton^58^ which revealed that they were under stress throughout Covid-19. According to the findings of the DASS-21, 3 (10%), 18 (60%), 4 (13%), 3 (10%), and 2 (6%) of the undergraduates displayed signs of normal, mild, moderate, severe, and extremely severe stress status, respectively^58^.

Another study of 384 undergraduates of both state and non-state universities/higher educational institutes reported 41% severe and 36% moderate stress levels due to the pandemic (during the year 2020) similar to the current evidence^40^ and another study of medical students^64^. It was also found that online learning experience and university workload affected many undergraduates during the Covid-19, and experienced more challenges with the information technology capabilities/infrastructure (50%) and increased workload (75%) which was a novel experience for the new entrants and majority of students who had economic hardships^40^. They did not discuss the influence of any socio-demographic characteristics on stress and found that new challenges and difficulties in carrying out day-to-day activities affected their stress. Further, they were more distressed about semester grades and how the class would impact their future^40^ and career opportunities. The start and end dates of degree programmes and postponements of planned examinations in educational settings may be influenced by the prevalence of certain stresses due to unexpected delays in educational activities.

According to a study by Galhenage et al.^62^, 78.2% of medical students were worried and had stress levels higher than those of working doctors, which was higher than the findings at the time of Covid-19. Distressed medical students were concerned about their own and their loved ones' health. Like doctors, those undergraduates believed that stress interfered with their everyday activities and that they had a high risk of getting Covid-19 (63.5%). Additionally, half of the students stated that while learning about Covid-19 was generally beneficial, it also led to more distress (24%)^62^. Before the pandemic, Sri Lankan research (N = 327) indicated that 40.4% of medical undergraduates had serious psychological distress^46^ while a much higher level of distress (78.2%) occurred due to the Covid 19. Among 527 medical undergraduates, the reported prevalence of anxiety and stress was 34% and 24.7%^63^. Possible explanations for these findings include that medical undergraduates being directly exposed to the clinical settings than other undergraduates would be a cause of having higher stress scores than our findings although we had not discussed about exposure status of studied undergraduates.

As evidenced, medical students had 9.6% depressive symptoms due to Covid-19 according to the DASS-21^61^. According to another study (N = 208), 40% of undergraduates reported mild to severe depressive symptoms and only 3% had severe symptoms^59^, and 40.8% of medical students reported depressive symptoms in a study of 527 undergraduates^63^. Slightly similar findings were reported in a previous study done among undergraduates from Sri Lanka, India, and Russia; 11.58% of students had elevated depressive symptoms due to Covid-19^7^. A low level of depression was reported during the study period among nursing undergraduates who studied at KIU, Sri Lanka; the revealed prevalence of depression was 7%^65^. When considering previous studies done in Western countries, undergraduates of the current study and other studies reported a considerably higher level of stress, anxiety, and depressive symptoms which may be due to new experiences with the pandemic (e.g., some countries have been exposed to H1N1, SARS, Bird’s flu, etc.) such as lock-down, quarantine, precautionary measures, and rising of cases and deaths globally. It has caused disruptions of ongoing physical education which had not been experienced by Sri Lankans due to a disease condition. Thus, adolescents and youth in Sri Lanka have experienced significant psychosocial stressors, making them more vulnerable to developing psychological morbidity due to the current crisis as reported in studies^12,66^.

A recent study conducted among 100 undergraduates of the same university, the UoR in Sri Lanka from December 2021-January 2022 revealed environmental factors and other associated factors of stress and psychiatric illnesses; 68% of undergraduates were psychologically distressed and 50% of the students were moderate to severely distressed^67^ similar to the current results. Reported main environmental factors that caused high stress were lack of time for leisure/sports activities (40–50%), following Covid-19 (30–40%), and issues related to online learning and evaluation (30%)^67^. However, they had studied many more reasons than the current study which focused on Covid-19 disease itself. Before Covid-19, 51.1% of nursing undergraduates at the University of Peradeniya, Sri Lanka had mild to extremely severe depressive symptoms same as our findings although not done during the Covid-19 period^68^. This argument was proved by the authors in Sri Lanka^12^ who stated that Sri Lankan adolescents usually have experienced higher levels of psychosocial stressors. As previously reported, student’s age, academic year, satisfaction with the nursing program, physical well-being elements, potential stresses, self-rated physical health, and self-rated mental health were all related to depressive symptoms^68^. Therefore, differences among psychological disturbances could occur due to socio-cultural, religious, and ethnic differences, and due to the usage of various scales/tools (e.g., 21-DASS, PHQ-9, GAD, SAS, CES-D scale, etc.) to measure stress, anxiety, and depressive symptoms, which might affect the outcome.

### Factors that influence stress, anxiety, and depressive symptoms and correlations between stress, anxiety, and depressive symptoms

Several factors associated with stress, anxiety, and depressive symptoms were found in the current study. Age and type of faculty were significantly associated with three types of psychological disturbances. Gender was significantly influenced by anxiety and depressive symptoms while depressive symptoms were significantly influenced by academic year. As reported in the current study, each psychological problem caused increased stress, anxiety, and depressive symptoms among undergraduates of the UoR.

As mentioned earlier in a study by Galhenage et al.^62^, medical students had higher levels of stress, and getting more information about the disease also increased their stress. When considering the medical undergraduates of the current study, they did not study about such a risk. However, the type of faculty or type of undergraduates had some influence on stress, anxiety, and depression in the current findings, respectively (*p* < 0.001*; p* < 0.05*; p* < 0.001). Medical undergraduates/FoM showed an impact on depressive symptoms while a higher number of humanities undergraduates/FoHSS had shown an impact on stress, anxiety, and depressive symptoms which may be due to low exposure to contagious disease or less awareness about the disease compared to the medical undergraduates. As another study of medical students in Sri Lanka in that period revealed, some factors of depression were the presence of economic difficulties (*p* < 0.05); previous contact with psychiatric services (*p* < 0.01); the presence of medical/surgical impairment); anxiety [e.g., previous contact with psychiatric services (*p* < 0.001)]; and stress, e.g., perceived lack of support from the university administration and difficulty accessing internet facilities, previous contact with psychiatric services (*p* < 0.001); presence of medical/surgical impairment]^61^. Another study’s findings were also similar showing depression (*p* < 0.01), anxiety (*p* < 0.05), and stress (*p* < 0.01) were significantly higher in medical students with a history of psychiatric disorders^63^ which was not studied in the current study. A working experience of nursing undergraduates who studied at KIU, Sri Lanka had a significant association with depression (*p* < 0.001), anxiety (*p* = 0.006), and stress (*p* = 0.011) and was further evidence for the type of undergraduates^65^ who were exposed to the clinical settings as medical students during their training period.

Female undergraduates in the current study had a significant impact on stress, anxiety, and depressive symptoms than their male counterparts similar to the previous medical students’ study^61,64^ and are similar in most Western studies^33,34^. However, gender significantly had an impact on depression (*p* < 0.05) and anxiety (*p* < 0.05). The Covid-19 epidemic caused psychological disturbances in 59% of female students compared to 49% of male students and stress, anxiety, and depressive symptoms were more common in female students^5^. A possible explanation for female undergraduates being more susceptible to psychological stressors compared with male undergraduates is that extra responsibility relates to caregiving activities and engaging with family members during the pandemic^33,34^. Our female undergraduates are largely socialized and capable of doing many duties and home activities simultaneously as explained by another study which states that girls played different roles within the family^53^. Also, their self-confidence about the disease condition being impacted by their low nutrition levels, low income, etc., during that period, may have contributed to higher distress, anxiety, and depression levels. Although women had more depression, men had a significant risk of severe depression symptoms (*p* < 0.05)^33^. A study by Rohanachandra et al.^63^ did not report any gender differences in anxiety, depression, or stress; these inconsistencies may be related to cross-cultural differences and unique traditions as suggested by Rogowska et al.^54^.

Further, older/senior undergraduates showed a significant influence on stress, anxiety, and depressive symptoms than younger participants as found in the current study (*p* < 0.001) while age was associated with the level of depression among nursing undergraduates at KIU (*p* = 0.016)^65^. It was discovered that being younger was a protective factor against experiencing depression symptoms^33^. A study of medical students reported the reason for having higher stress among senior students might be due to the difficulty in making career choices, and opinions, and a lack of self-confidence in fitness for future clinical practice^69^. In contrast to the current findings, younger and distance learners in OUSL (e.g., Humanities and social sciences, Natural science, Engineering, Education and Health sciences faculties) had reported significantly higher levels of stress, anxiety, and depressive symptoms although they used 21-DASS scale like the current study^39^. However, only depressive symptoms had a significant impact by the academic year in the current study (*p* < 0.05). As in other findings, first-and second-year medical students had higher depressive symptoms compared to the fourth year (*p* < 0.05)^63^; the final years reported additional psychological distress and burnout than first -fourth, and fifth-year students evidenced lower positive mental health than first year students^64^ which could be a reason of increased responsibilities in the final clinical years of training.

Some studies found no significant difference in stress, anxiety, and depression among undergraduates regarding age^7^, gender^7,63^, and type of degree^7^. Additionally, some nations mentioned reasons for lesser mental illnesses as developed healthcare systems and the wide range of emergency medical treatments and facilities for Covid-19. As a result, undergraduates in these nations were less concerned about safety precautions and other Covid-19-related resources than others^33,53^ compared to the condition in our country. Additionally, putting strict health protocols into practice, such as using face masks and hand washing, telling people to "stay at home," and closing public places, may have increased their sense of security although it increased their psychological problems. However, during that time, longer lockdowns and increased Covid-19 cases/deaths led to higher levels of stress and anxiety because of the closure of religious sites like mosques and fully covered female attire (e.g., Abhaya)^34^ mostly relevant to Arabic countries/Islamic ethnic groups but related to our nations as well.

Furthermore, our study discovered a significant positive correlation between depression, anxiety, and stress (depressive symptoms-anxiety: r = 0.646, *p* < *0*.001; depressive symptoms-stress: r = 0.868, *p* < 0.001; and anxiety-stress: r = 0.786, *p* < 0.001). In 2016, a survey conducted among nursing undergraduates in Sri Lanka evidenced that most similar correlations are in line with the current findings. The reported significant positive relationships were; anxiety and depression (r = 0.689, *p* < 0.001), anxiety and stress (r = 0.785, *p* < 0.001), and depression and stress (r = 0.763, *p* < 0.001)^68^; this significant relationship also suggests a reduced risk for psychiatric illness in undergraduates. It means that if there is no risk of having a higher level of stress, they are prone to low anxiety and/or depression in other ways^68^. These negative emotional sensations can lead to poor mental health, which impairs learning and limits academic success. Hence, when the undergraduates are overburdened, it may cause further emotional instabilities and deplete them physically and mentally^70^. As explained by Rogowska et al.^54^ it could be summarized that if students worry too much about their health, their level of stress may increase, which may be an additional source of anxiety, and they would end up with severe stress and depression which may need medical assistance.

Considering a very vulnerable population, university undergraduates face a very important transition from high school to university^71,72^ which is a vital event in late adolescence associated with structural and social changes that influence relationships, routines, assumptions, and individualization in roles such as moving out of home, becoming independent from parents, searching/meeting new friends, facing a new guidelines/responsibility and higher workload of education. Those are some critical events as evidenced during their transition which led to negative consequences such as loneliness, stress, anxiety, depression, etc. in their life^71,72^ as studied in this study which need more attention by both parents and university academics.

### Original contribution and relevance

However, when compared to the Western Province of Sri Lanka, no studies have been published on the psychological problems due to the Covid-19 outbreak in undergraduates in the Southern Province. Therefore, it was better to find out the impact of the disease on the education of undergraduates at the UoR which is the only state university in Southern Sri Lanka. Further, we were able to assess the psychological impact of different five faculties rather than focusing only on medical undergraduates as done by most researchers in Sri Lanka. Universities must develop and implement effective screening procedures to closely monitor undergraduates’ exposure to different stressors and mental health adjustment and to increase the academic performances and student support services of undergraduates in future pandemics. This research will be vital for higher educational sectors and policymakers to create welfare programmes and psychological support services for future undergraduates. Further, this study has important implications for university administration in identifying, treating, and preventing mental health problems among undergraduates during acute, large-scale stressors like infectious disease outbreaks or natural disasters.

### Limitations

Due to the institutions being closed at the time of the survey, data were gathered using an online questionnaire, which prevented participants from asking questions regarding the survey or its components. A lack of availability of internet facilities may have predisposed the response rates of students. Additionally, the survey did not allow the contributors to seek explanations about the survey or items in the questionnaire. This study used a cross-sectional design, which makes it impossible to determine the cause and effect/causal relationship among variables. Data were gathered from a single university in Sri Lanka; respondents' impressions may differ depending on unique aspects of the setting, degree programmes, and the curriculum. Also, some variables such as income, ethnicity, religion, and academic/examination workload were not included in this study and may have some correlations. Because the sample was representative of the population of university undergraduates, the results were rather reliable, but they cannot be generalized to other settings and the whole of Sri Lanka. Yet, the current study incorporated standardized scales to examine the stress, anxiety, and depression symptoms.

### Clinical implications

These results of the current study are useful in detecting the need for psychological support programmes to find undergraduates with a high risk of psychological disturbances. Further, it would be better to find reasons for higher levels of psychological disturbances among undergraduates studying in the Southern province than the Western province studies while the Southern province had lower severity than the Western province. Universities must plan short-term and long-term psychological amenities for undergraduates and inspire undergraduates to get support from mental health professionals when essential. Further, examination/investigations and a clinical diagnosis by a psychiatrist may be required for undergraduates who scored extremely severe levels for stress and depressive symptoms. Mental health support strategies specifically need to be focused on undergraduates who are their older ages, females, studying in non-health-related degree programmes, and first-final years. The undergraduates with diagnosed mental problems, such as depression or anxiety disorder, should be under constant monitoring of psychological and psychiatric services^73^.

## General conclusions

There has been an increase in psychological problems among undergraduates due to the Covid-19 outbreak. Overall, a significant level of stress, anxiety, and depressive symptoms were reported among undergraduates selected from five faculties, at the University of Ruhuna in Sri Lanka. Age, gender, and type of faculty significantly influenced the level of stress, anxiety, and depressive symptoms while academic year was significantly correlated with depressive symptoms. Further, stress, anxiety, and depression were significantly and positively associated with each other aspect.

Early detection of psychological issues and their associated factors is essential. Furthermore, parents should be stimulated to generate a friendly and positive family environment for undergraduates without imposing pressure on their future academic careers. It is necessary to develop stress management strategies including coping mechanisms and expand counseling opportunities for undergraduates. To prevent mental health problems among undergraduates, it is essential to plan effective programmes and policies. The government and universities should collaborate to provide quick, accurate, and cost-effective psychological support to undergraduates to reduce the escalating mental health issues.

### Strengths

A strength of our study was that this is one of the few studies that examine the self-reported prevalence of stress, anxiety, and depression among different university undergraduates in Sri Lanka and the only study conducted in the Southern Province, not just focusing especially on medical degree programmes, etc., but vital for observing all stakeholders of the university population and as community health research. Another positive factor of this study was the high response rate comparatively. This study was conducted during the Covid-19 pandemic and provided us further awareness of the possible increase in psychological health problems that undergraduates might face due to different reasons: health and educational concerns; disruption of normal life; economic challenges; and concerns about the future causing a higher level of stress, anxiety, and depressive symptoms especially by those who were in the Southern province who were not much affected as those in the Western province of Sri Lanka. Nevertheless, the usage of a validated screening tool was measured as a cost-effective method to explore the situation in general and the first time that we applied many components of the research process online which was not experienced earlier.

## Recommendations for policymakers and stakeholders of higher education

University/government/authorities should plan short-term and long-term goals and/or psychological services for undergraduates. Short-term goals are;

1. Immediate actions need to be taken to uplift the psychological well-being of undergraduates.
2. The university authorities must consider the associated factors for increased psychological disturbances and must expand resources such as online/distance learning facilities, infrastructure, supervision, and academic instruction when needed.
3. Take action to provide financial assistance and authorities should expand facilities/resources for providing resources for sports, music, and cultural events as suggested by another author^67^.
4. There is also a need to raise awareness of the mental health of undergraduates through training and educational programmes that promote their mental health in future pandemics.
5. Additionally, it is crucial to create efficient interventions and strategies to reduce psychological health difficulties among university undergraduates. Authorities should emphasize making sure there are enough facilities and resources.
6. To compare the psychological health of university undergraduates across Sri Lanka, more research is required using a validated scale^12^; undergraduates from various faculties, university settings/types (state and non-state), and locations should be included in future studies.
7. This research focused on the start of Covid-19 and the quarantine once the psychological level may be at its highest level; longitudinal and qualitative surveys are necessary to identify changes in undergraduates' mental health over time (to assess the relationship between different associated factors using a wide range of explanatory variables that can explain psychological disturbances). Further, in-depth interviews/discussions are recommended to explore such issues.
8. Empower the existing programmes, and educational professionals to implement measures to improve emotional well-being and professional organizations.
9. Reinforce the capacity of the mental health helpline/chatline 1926 by the National Institute of Mental Health to provide for children/adolescents, raise awareness of this service^12^, and encourage undergraduates to get support from mental health professionals when necessary.
10. Promote awareness about mental health promotional activities using electronic and printed media.
11. Make youth-friendly informative videos and posts on social media about psychological well-being, reconciliation, social responsibility, etc^12^.
12. Conduct mental health promotional activities and improve awareness about coping strategies (e.g., adaptive/constructive/problem-based coping strategies and maladaptive/destructive/emotional-based coping strategies) of undergraduates through available departments of psychiatry and psychology already established in all state universities in Sri Lanka^12^.
13. Both the government and universities must plan collective strategies to find a solution to academic delays, academic and professional uncertainty, and financial insecurity due to unexpected circumstances.

Long-term goals are;

1. Universities should establish all-inclusive online-based educational programs for undergraduates living in remote areas with or without devices and provide scholarships or student loans who are in need.
2. If such social distancing measures are implemented in future diseases, telehealth, digital mental health services, and tele-counseling programmes can be used as strategies (economy-oriented psychological support) to make these vulnerable people.
3. Universities should actively assess the mental illness of undergraduates at various stages of their academic careers and set up and announce helplines and other forms of support.
4. The Department of Psychiatry or Medical Centre in the Universities should take the initiative and take the lead in developing suitable psychological support services within the faculty since it plays a vital role in providing this information; authoritative personnel/medical officials could offer to be freely available for in-person or online discussions with undergraduates and, if necessary, to direct them to alternative mental health services. Additionally, they can conduct meetings about the effects of Covid-19 on mental health and promote the help-seeking behavior of undergraduates.
5. Offer more human resources (e.g., medical and nursing officers, and allied health professionals) and monetary facilities to government child and adolescent mental health services^12^.

## Data Availability

The data analyzed throughout the current study are not publicly available due to ethical reasons. The survey data will be made available on reasonable request to the corresponding author.
